# Hepatic Metastasis of Meningioma Treated with Partial Hepatectomy: A Case Report

**DOI:** 10.70352/scrj.cr.26-0401

**Published:** 2026-10-16

**Authors:** Yuki Hirase, Haruhi Fukuhisa, Akihiko Sakamoto, Masahiko Sakoda, Sumika Matsukita, Takuya Hiwaki, Shigeho Maenohara

**Affiliations:** 1 Department of Surgery, Kagoshima Kouseiren Hospital, Kagoshima, Kagoshima, Japan; 2 Department of Pathology, Kagoshima Kouseiren Hospital, Kagoshima, Kagoshima, Japan; 3 Department of Hepatology, Kagoshima Kouseiren Hospital, Kagoshima, Kagoshima, Japan

**Keywords:** hepatic metastasis, meningioma, hepatectomy

## Abstract

**INTRODUCTION:**

Meningioma is one of the most common central nervous system tumors; however, metastatic lesions are extremely rare. Particularly, hepatic metastasis from meningioma is uncommon, and its diagnosis and optimal management remain undefined.

**CASE PRESENTATION:**

A 70-year-old man with a history of left parietal meningioma resection in 2011 and gamma knife therapy for recurrence in the left lateral ventricle in June 2025. A PET-CT scan performed for cancer screening in June 2025 revealed abnormal fluorodeoxyglucose uptake in segment 8 of the liver, with a maximum standardized uptake value of 6.2. CT, and MRI revealed multiple hepatic masses. All tumor markers were within normal ranges. A percutaneous liver biopsy indicated a spindle cell tumor; however, the diagnosis remained inconclusive. For diagnostic and therapeutic purposes, partial hepatectomy of segments 6 and 8 and cholecystectomy were performed. Histopathological examination revealed proliferating spindle-shaped tumor cells arranged in fascicles. Immunohistochemical staining was positive for vimentin, epithelial membrane antigen, and somatostatin receptor 2, confirming the diagnosis of hepatic metastasis from meningioma. The postoperative course was uneventful, and the patient was discharged in good condition.

**CONCLUSIONS:**

Hepatic metastasis from meningioma is extremely rare; however, it should be considered in the differential diagnosis of hepatic tumors in patients with a history of meningioma. Surgical resection is valuable in establishing a definitive diagnosis and achieving local disease control.

## Abbreviations

AFP
: alpha-fetoprotein
CA19-9
: carbohydrate antigen 19-9
CEA
: carcinoembryonic antigen
EMA
: epithelial membrane antigen
FDG
: fluorodeoxyglucose
HCC
: hepatocellular carcinoma
PIVKA-II
: protein induced by vitamin K absence or antagonist-II
S6
: segment 6
S8
: segment 8
SSTR2
: somatostatin receptor 2
SUVmax
: maximum standardized uptake value
WHO
: World Health Organization

## INTRODUCTION

Meningioma is the most common primary tumor of the central nervous system, accounting for approximately 30% of all intracranial neoplasms.^1)^ Most cases are classified as WHO grade I, characterized by slow growth and a favorable prognosis following surgical resection. However, a subset of cases demonstrates recurrence or malignant transformation. In particular, WHO grades II (atypical meningioma) and III (anaplastic meningioma) are associated with higher rates of local recurrence and distant metastasis.^2)^

Distant metastasis from meningioma is extremely rare, occurring in less than 1% of all cases.^3)^ The most common metastatic sites are the lungs and bones, followed by the lymph nodes, liver, and kidneys.^4)^ The potential mechanisms of dissemination include surgical seeding, hematogenous spread via venous sinuses or dural veins, and lymphatic spread; however, the exact pathways remain unclear.^5)^ Hepatic metastasis from meningioma is exceptionally uncommon, with only a few dozen cases reported worldwide.^6)^

Clinically, meningioma is generally regarded as a benign intracranial tumor, and postoperative follow-up rarely predicts recurrence or distant metastasis. Therefore, when a hepatic mass appears during follow-up, metastatic meningioma is seldom considered in the differential diagnosis.^7)^ In most cases, preoperative differentiation from other hepatic tumors, such as HCC, metastatic adenocarcinoma, hemangioma, or cholangiocarcinoma, can be challenging. Histopathological evaluation, including immunohistochemical analysis, is essential to establish a definitive diagnosis.^7)^

Herein, we report a rare case of hepatic metastasis from meningioma in a patient with a history of intracranial meningioma resection and subsequent gamma knife treatment for recurrence. The hepatic lesion was successfully resected, and the diagnosis of metastatic meningioma was confirmed histologically. This case illustrates an extremely rare pattern of meningioma metastasis and underscores the diagnostic and therapeutic value of surgical resection in such cases.

## CASE PRESENTATION

A 70-year-old man was referred after PET-CT, performed as part of a cancer screening in June 2025, revealed abnormal FDG uptake in S8 of the liver, with a SUVmax of 6.2 (**Fig. 1A**). CT and MRI revealed a 30-mm tumor in S8 (**Fig. 1B**, **1D**) and a 20-mm tumor in S6 of the liver (**Fig. 1C**, **1E**). All serum tumor markers, including AFP, CEA, CA19-9, and PIVKA-II, were within normal ranges.

**Fig. 1:**
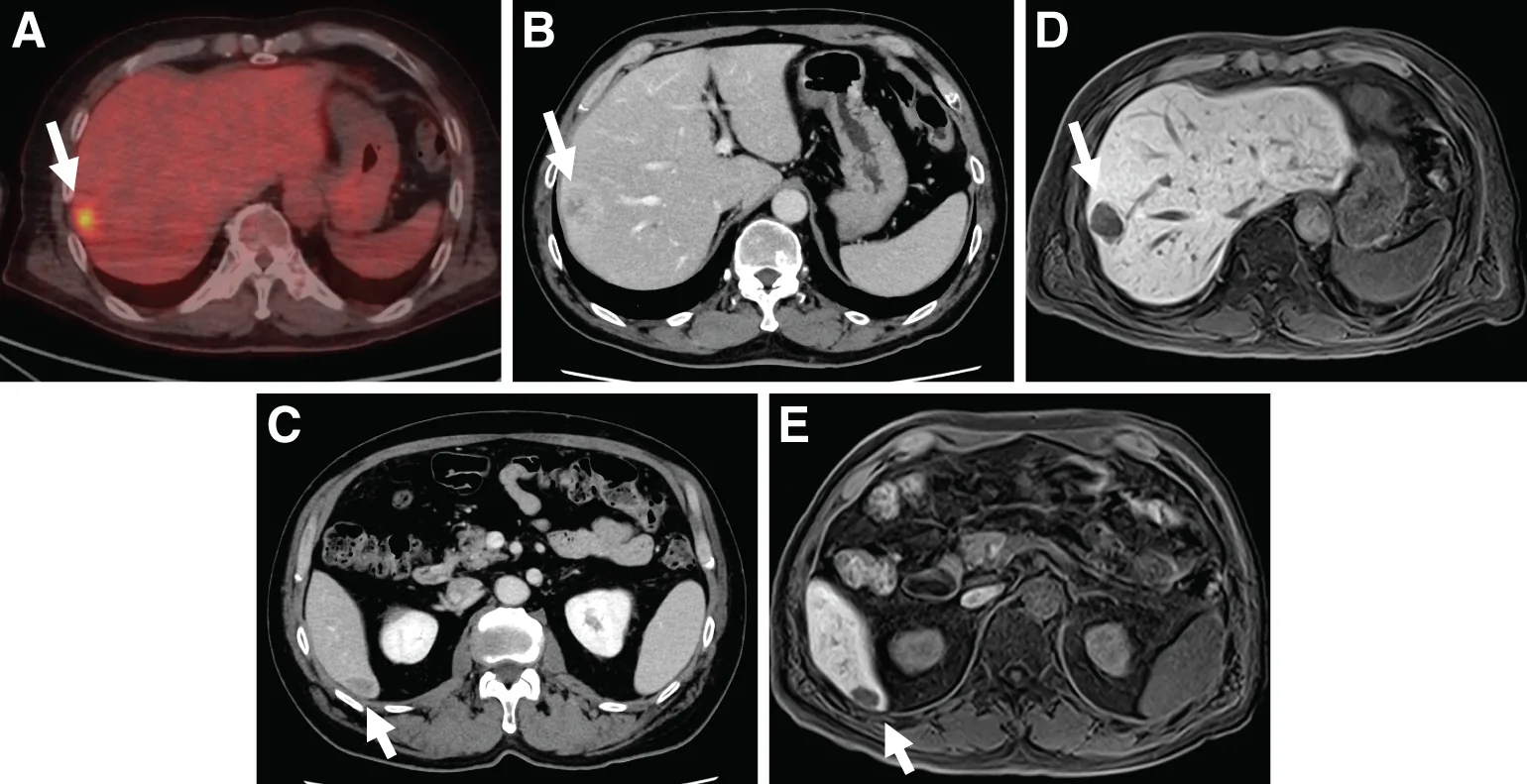
Composite radiological findings. (**A**) PET-CT showing abnormal FDG uptake in S8. (**B**, **C**) Enhanced CT showing the hepatic lesions in S6 and S8. (**D**, **E**) MRI showing the corresponding lesions in S6 and S8. FDG, fluorodeoxyglucose; S6, segment 6; S8, segment 8

The patient had a history of left parietal meningioma (WHO grade I) resection in 2011 and gamma knife therapy for recurrence in the left lateral ventricle in June 2025. The overall clinical course is summarized in **Fig. 2**. A percutaneous needle biopsy of the hepatic tumor was performed by the hepatology department in June 2025, which revealed a spindle cell tumor. Based on the pathology results, angioleiomyoma, schwannoma, and leiomyoma were considered possible diagnoses. Additionally, given the patient’s history of treatment for a meningioma, metastasis from a meningioma was also considered as a differential diagnosis. Subsequently, the patient was referred to our surgical department for diagnostic resection. An open partial hepatectomy and cholecystectomy were performed.

**Fig. 2:**
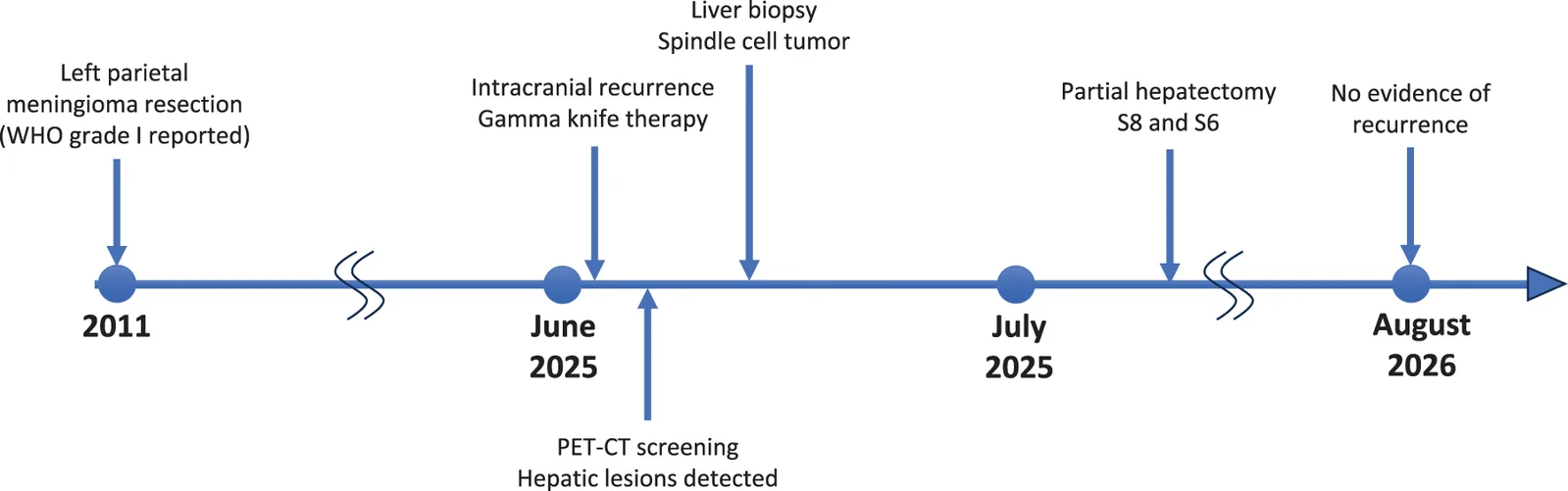
Timeline of the clinical course from initial intracranial meningioma resection to hepatic metastasectomy.

During surgery, no new hepatic lesions other than those identified preoperatively were observed. The gallbladder was first removed, and a C-tube was inserted through the cystic duct. The right lobe of the liver was mobilized to fully expose the S8 lesion, which was resected with a surgical margin of approximately 10 mm. Subsequently, the S6 lesion was resected. A bile leak test using the C-tube confirmed the absence of bile leakage. The total operative time was 3 hours and 47 minutes, with an estimated blood loss of 165 mL, and no transfusion was required. The postoperative course was uneventful, and the patient was discharged on POD 19. He remains under follow-up at our department and the neurosurgery department. At the latest follow-up, 13 months after hepatectomy, he was alive without evidence of intrahepatic recurrence, extrahepatic metastasis, or progression requiring additional treatment.

Gross examination of the resected specimen revealed well-circumscribed, solid, whitish tumors measuring 35 × 25 × 21 mm in S8 and 20 × 14 × 12 mm in S6 (**Fig. 3**). Microscopically, both tumors consisted of spindle-shaped cells arranged in fascicular and whorled patterns. There were more than 4 mitotic figures per 10 high-power fields. Immunohistochemical staining was positive for vimentin, EMA, and SSTR2, with a Ki-67 labeling index of 11% (**Fig. 4**). Based on these findings, the final diagnosis was hepatic metastasis of a meningioma.

**Fig. 3:**
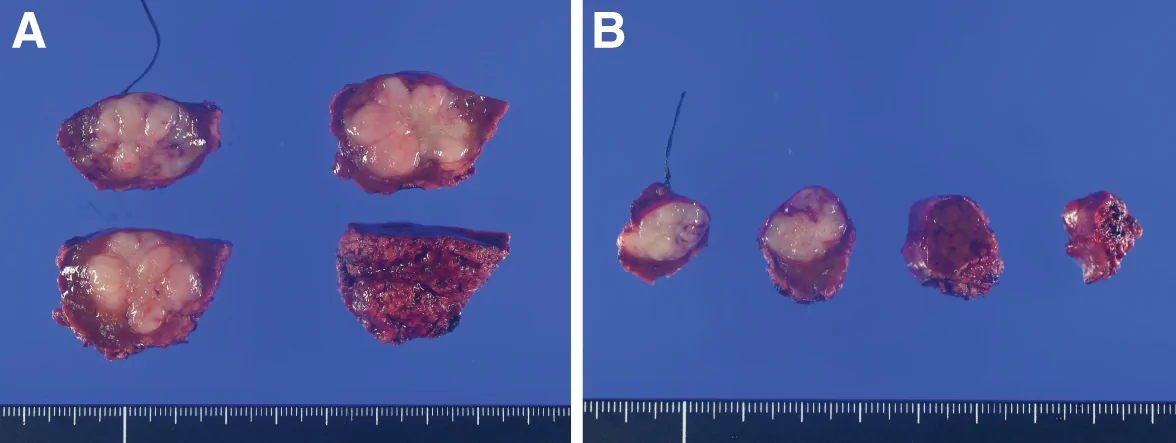
Macroscopic findings of the resected liver lesions in S8 (**A**) and S6 (**B**). S6, segment 6; S8, segment 8

**Fig. 4:**
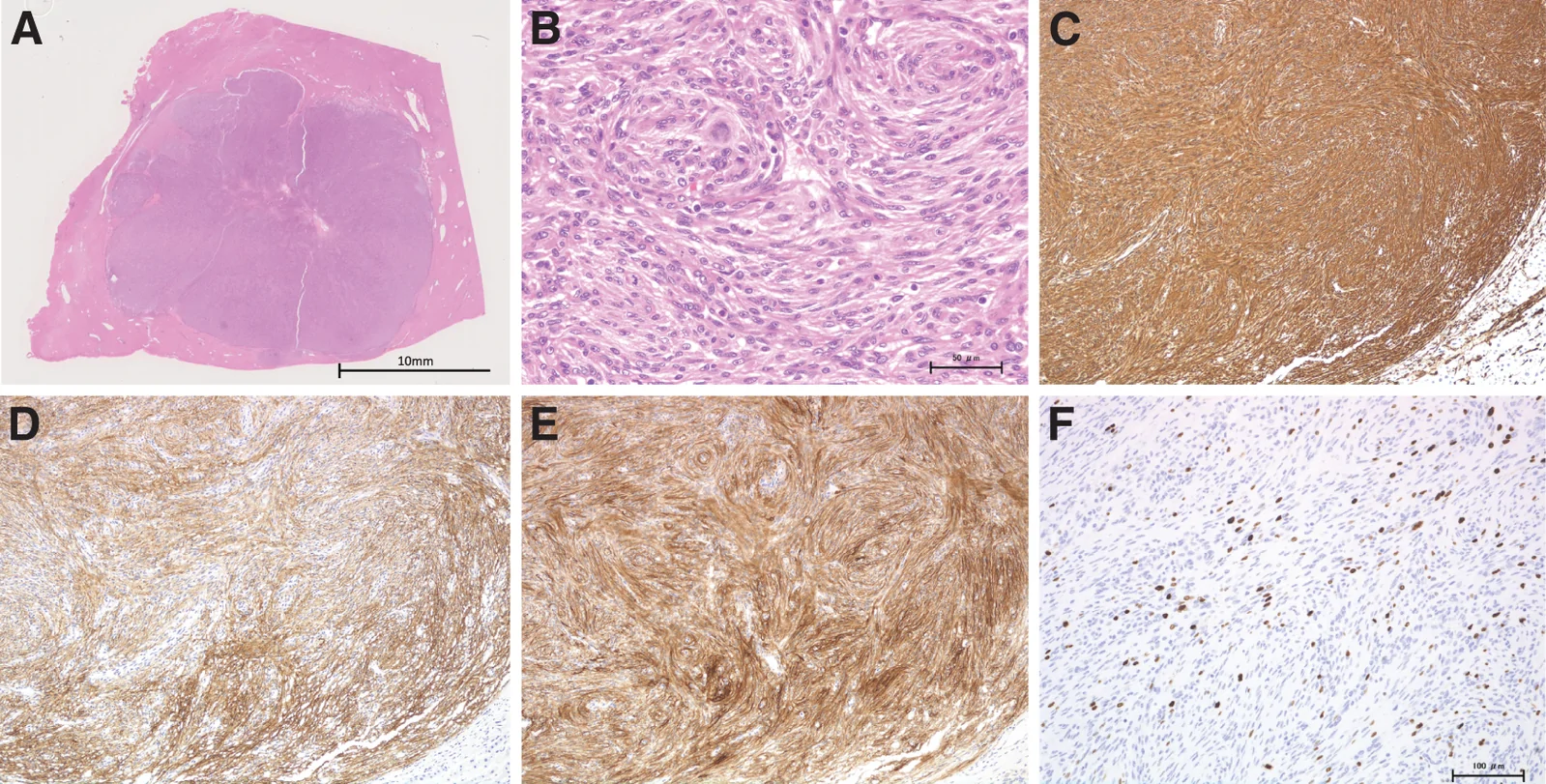
Histopathological and immunohistochemical findings of the hepatic lesion. Hematoxylin and eosin staining shows spindle-shaped cells arranged in fascicular and whorled patterns (**A**: low magnification; **B**: high magnification). Immunohistochemical staining for vimentin (**C**), EMA (**D**), and SSTR2 (**E**) is strongly positive. The Ki-67 labeling index is 11% (**F**). EMA, epithelial membrane antigen; SSTR2, somatostatin receptor 2

We attempted to compare the surgical specimen from the meningioma surgery he underwent at age 56 with the current specimen, but since the original pathology specimen and pathology test results from that time were no longer available, we were unable to make a direct comparison.

## DISCUSSION

Although meningioma is a relatively common tumor of the central nervous system, distant metastasis occurs in less than 1% of all cases and is therefore extremely rare.^3)^ The lungs and bones are the most frequent metastatic sites, while reports of hepatic metastasis are even more uncommon.^4)^ In the differential diagnosis of hepatic tumors, primary HCC and metastatic carcinomas originating from the colon, stomach, or pancreas are typically considered. However, in patients with a history of meningioma, hepatic metastasis should also be considered.

In the present case, PET-CT revealed abnormal FDG uptake with an SUVmax of 6.2. Radiological findings showed hepatic masses that did not exhibit typical features of HCC or intrahepatic cholangiocarcinoma. Furthermore, all tumor markers, including AFP, PIVKA-II, CEA, and CA19-9, were within normal ranges, making preoperative diagnosis difficult. Although percutaneous biopsy revealed a spindle cell tumor, a definitive diagnosis could not be established. Immunohistochemical analysis of the resected specimen showed strong positivity for EMA, vimentin, and SSTR2. As SSTR2 is recognized as a useful marker for identifying meningioma among spindle cell tumors, a final diagnosis of hepatic metastasis from meningioma was made.

Several mechanisms have been proposed for the metastatic spread of meningioma, including 1) surgical dissemination, 2) hematogenous spread, and 3) lymphatic spread. Previous reports have suggested that metastasis tends to occur in patients with recurrent disease or those who have undergone radiotherapy. In this case, the patient had a history of recurrence and had received gamma knife therapy, which may have contributed to the development of distant metastasis.^8)^

Currently, there are no standardized treatment strategies for metastatic meningioma. However, surgical resection is considered effective for local control and definitive diagnosis.^4)^ In this case, surgical removal of the hepatic lesions achieved both diagnostic confirmation and satisfactory local control. Nevertheless, given the presence of distant metastasis, the possibility of intrahepatic recurrence remains high.^7)^ In such cases, repeat hepatectomy or local therapies such as radiofrequency ablation and radiotherapy may be considered. Moreover, since many meningiomas express SSTR2, recent studies have reported the therapeutic efficacy of somatostatin analogs and peptide receptor radionuclide therapy.^9)^ These treatment modalities may represent potential options for patients with positive surgical margins or recurrent disease.

Hepatic metastasis from intracranial meningioma remains exceptionally uncommon. A 2021 literature review identified 24 patients with hepatic dissemination; 14 had WHO grade II/III tumors, whereas 9 had WHO grade I tumors, and approximately half had multiple hepatic lesions. Surgical resection or biopsy was frequently used for diagnosis and local control, while systemic therapy or observation was selected in patients with multifocal or disseminated disease.^6)^ The clinical characteristics and management of previously reported cases, together with the present case, are summarized in **Table 1**. More recent reports have likewise described long intervals between treatment of the intracranial tumor and hepatic metastasis, including 6–12 years in several cases, and successful hepatectomy has been reported in selected patients with resectable disease.^10–15)^ These observations suggest that hepatic metastasis may occur even after a long clinically indolent course and even from histologically low-grade meningioma. Compared with previously reported cases, the present case is characterized by 2 resectable hepatic lesions, a long interval of approximately 14 years from the initial intracranial resection, and histological features of atypical meningioma in the liver despite the original tumor having been reported as WHO grade I. Because the original pathological material was unavailable for re-review, whether this discrepancy represents sampling limitations of the primary tumor, biological progression over time, or true malignant transformation cannot be determined. Nevertheless, the long interval and change in pathological grade support the need for prolonged clinical awareness of extracranial disease in patients with recurrent meningioma.

**Table 1:** Comparative review of reported hepatic metastases from intracranial meningioma

Report	Age/sex	Primary WHO grade	Previous cranial treatment	Interval to hepatic metastasis	Hepatic lesions	Treatment for hepatic disease	Outcome
Beutler et al., 2020^10)^	71/F	I	Multiple resections for recurrent disease; exact n NR	~7 year	3; largest 5.5 cm	Biopsy; bevacizumab/systemic therapy	Referred for systemic therapy
Limarzi et al., 2020^11)^	68/F	II	1 partial resection + gamma knife	6 year	Solitary right-lobe; size NR	Segmentectomy	Alive, recurrence-free at report
Shimokawa et al., 2020^12)^	58/F	II	1 resection before hepatic metastasis	9 year	Solitary; size NR	Mass resection	Good course; later intracranial reoperation
Attuati et al., 2019^6)^	61/F	II	NR	NR	NR	Liver biopsy	NR
Unterrainer et al., 2019^6)^	43/F	II	NR	NR	Multiple; size NR	NR	NR
Obiorah and Ozdemirli, 2018^7)^	54/F	I	No known cranial surgery before liver diagnosis	Synchronous/occult primary	Solitary left-lobe; size NR	Partial hepatectomy	Cranial primary subsequently identified
Villanueva-Meyer et al., 2018^6)^	52/F	II	NR	NR	Solitary S4b; size NR	Liver biopsy	NR
Kessler et al., 2017^6)^	65/M	III	NR	NR	Right-lobe; size NR	Hydroxyurea	NR
Kessler et al., 2017^6)^	49/M	II	NR	NR	Number/size NR	NR	NR
Forest et al., 2014^6)^	80/F	II	NR	NR	Right-lobe; size NR	Biopsy + excision	NR
Forest et al., 2014^6)^	68/M	I	NR	NR	Multiple; size NR	Liver biopsy	NR
Lanfranchi and Nikpoor, 2013^6)^	74/M	II	NR	NR	Multiple; size NR	NR	NR
Lambertz et al., 2011^6)^	65/F	II	NR	NR	Number/size NR	Hydroxyurea + biopsy	NR
Taieb et al., 2011^6)^	30/M	II	NR	NR	Multiple; size NR	Observation	NR
Rampurwala et al., 2011^6)^	81/F	I	NR	NR	12 lesions; size NR	Biopsy + observation	NR
Asghar et al., 2009^6)^	55/M	II	NR	NR	Multiple; size NR	Biopsy + ifosfamide	NR
Garcia-Conde et al., 2009^6)^	44/M	III	NR	NR	Multiple; size NR	Liver biopsy	NR
Khalbuss et al., 2005^6)^	71/F	NR	NR	NR	Multiple; size NR	NR	NR
Ku et al., 2005^6)^	47/F	I	NR	NR	Solitary right-lobe; size NR	Biopsy + resection	NR
Nabeya et al., 1998^6)^	60/M	Atypical/malignant	NR	NR	Solitary right-lobe; size NR	Biopsy + right hepatectomy	NR
Enam et al., 1996^6)^	73/F	Malignant features; grade NR	NR	NR	Right-lobe; size NR	Liver biopsy	NR
Ferguson and Flinn, 1995^6)^	37/M	I (angioblastic*)	NR	NR	1 right + multiple left-lobe lesions	Chemoembolization	Good clinical response
Jenkinson et al., 1987^16)^	38/M	I (angioblastic*)	NR	NR	1 right + multiple left-lobe lesions	Liver biopsy	NR
Akagi et al., 1974^6)^	33/F	I (angioblastic*)	NR	NR	Solitary left-lobe; size NR	Observation	NR
Higa et al., 2021^15)^	66/F	NR	2 resections (1998, 2012)	21 year from initial surgery to liver detection	3 (S6/S5/S2); sizes NR	Partial hepatectomies	NR
Feltracco et al., 2023^17)^	61/F	I	1 resection	~10 year	Solitary right-lobe; >20 cm	Right hepatectomy + cholecystectomy	Uneventful postoperative recovery
Borni et al., 2024^18)^	31/M	II	Multiple excisions; exact n NR	3 year	Multiple; largest 18 cm	Biopsy + chemotherapy	Favorable outcome reported
Present case	70/M	I primary; II features in liver	1 resection + gamma knife	~14 year	2 (S8/S6); largest 3.5 cm	Partial hepatectomy S8/S6	No recurrence at 13 month

Data were compiled from the 24-case review by Costea et al.,^6)^ supplemented by individual reports and cases published thereafter.^10–18)^

*Historical “angioblastic meningioma” overlaps with entities now classified separately, including solitary fibrous tumor/hemangiopericytoma; older reports should therefore be interpreted cautiously.

F, female; M, male; NR, not reported; S2, segment 2; S5, segment 5; S6, segment 6; S8, segment 8; WHO, World Health Organization

The management of hepatic metastasis should be individualized according to the number and distribution of liver lesions, the status of intracranial and other extracranial disease, and the patient’s general condition. Published cases include partial or major hepatectomy, biopsy followed by systemic therapy, transarterial treatment, radiotherapy, and observation.^6,10–16)^ In patients with technically resectable liver-limited or oligometastatic disease, hepatectomy can provide definitive histopathological diagnosis and durable local control; however, recurrence in the remnant liver or at other sites has also been reported. Therefore, continued systemic and intracranial surveillance is warranted even after complete hepatic resection.

## CONCLUSIONS

Hepatic metastasis from meningioma is extremely rare; however, it should be considered in the differential diagnosis when a hepatic mass is detected in patients with a history of meningioma. Surgical resection is valuable both diagnostically and therapeutically.

## DECLARATIONS

### Availability of data and materials

The datasets generated in this study are available from the corresponding author upon reasonable request.
